# Expression characteristic, immune signature, and prognosis value of EFNA family identified by multi-omics integrative analysis in pan-cancer

**DOI:** 10.1186/s12885-022-09951-0

**Published:** 2022-08-10

**Authors:** Zonglin Jiao, Xiao Feng, Yuqing Cui, Lei Wang, Junqing Gan, Yanbin Zhao, Qingwei Meng

**Affiliations:** 1 Department of Medical Oncology, Harbin Medical University Cancer Hospital, Harbin, China; 2 Department of Oncology, Central Hospital Affiliated to Shandong First Medical University, Jinan, China

**Keywords:** EFNA family, Pan-cancer, Tumor immune microenvironment, Tumor genesis, Multi-omics bioinformatics

## Abstract

**Background:**

EphrinA (EFNA) are Eph receptor ligands that regulate various disease processes. Nonetheless, the expression characteristics of EFNAs in pan-cancer, their relationship with tumor immune microenvironment, and prognostic value landscape remain unknown.

**Methods:**

A comprehensive landscape of EFNAs was created using various statistical data extracted from 33 cancers. Subsequently, we identified differential expression, genetic variations, potential function enrichment, tumor immune-related analysis, and drug sensitivity. Further, we investigated the clinical features and diagnostic prognostic value of EFNAs. RT-qPCR, western blot and immunohistochemistry (IHC) were used to validate the expression level and significant clinical value of EFNA5 in lung adenocarcinoma cell lines and tissues.

**Results:**

EFNAs were highly mutated in various cancers. Genomic and epigenetic alterations of EFNAs were observed in various tumors, where an oncogenic mutation in specific cancer types potentially affected EFNA expression. Moreover, tumor-derived EFNAs were significantly related to the tumor immune microenvironment, suggesting that they are promising therapeutic targets. The majority of EFNA family genes were significantly linked to patient prognosis. Eventually, EFNA5 was an independent prognostic factor in lung adenocarcinoma.

**Conclusion:**

In summary, EFNAs are crucial in tumor immune regulation, and EFNA5 is a prognostic marker in lung adenocarcinoma. Our findings provide new insights into EFNAs from a bioinformatics standpoint and highlight the significance of EFNAs in cancer diagnosis and treatment.

**Supplementary Information:**

The online version contains supplementary material available at 10.1186/s12885-022-09951-0.

## Introduction

In addition to radiotherapy and chemotherapy, treatments that increase anti-tumor immunity have become the standard of care for cancer patients in recent years [1–3]. Although targeted immunotherapies, including anti-CTLA4, anti-PD1/anti-PDL1, and CAR-T cell therapy have demonstrated notable efficacy, immunotherapy remains only effective for a subset of patients. Primary and acquired resistance must be addressed through a combination of multiple therapeutic approaches [4–7]. Therefore, the identification of suitable prognostic markers and therapeutic targets is important.

EphrinAs are ligands that bind to and interact with Eph receptors (Ephs), the largest subfamily of receptor tyrosine kinases (RTKs). The EphrinA ligand family has five members (Gene Symbol id: *EFNA1, EFNA2, EFNA3, EFNA4, EFNA5*) with a glycosylphosphatidylinositol anchor. Ephs/EFNAs are membrane-bound proteins requiring direct cell–cell interaction to activate downstream signals. Signal transduction is bidirectional, with forwarding signaling via Eph receptors and reverse signaling via ephrin ligands [8]. Ephs/EFNAs are implicated in several human physiological processes, including axonal orientation, cell adhesion and movement, synaptic development, as well as cell–matrix [9]. Meanwhile, many studies have focused on the link between Ephs/EFNAs, tumor genesis and progression, tumor behavior and features, as well as tumor microenvironment [10]. In recent years, Ephs have received significant research attention as one of the most promising immune-related drug targets [11].

Nonetheless, EFNAs could also modulate tumor development and immune microenvironment as essential Ephs ligands. EFNA1 overexpression in activated endothelium in vitro results in E-selectin, and VCAM-1 binding to leukocyte integrins [12], and EFNA1 stimulating CD4^+^ T cells promote the chemotaxis of stromal cell-derived factors and macrophage inflammatory proteins [13]. EFNA2 and EFNA3 have been linked to CD8^+^ T cells, Treg cells, and other immune cells [14]. Several recent studies have confirmed the role of EFNA4 in the progression of proliferation and metastasis of various cancers [15, 16]. Also, EFNA5 is implicated in several tumorigenic processes [17, 18]. Nevertheless, the immunological role, the expression level, and prognostic value of EFNAs in pan-cancer remain underexplored.

Herein, we comprehensively explored the genomic, transcriptome, epigenetic, immune microenvironment, clinical diagnosis, prognosis, and therapeutic value of the EFNA family in pan-cancer. IHC was used to validate EFNA5 as a critical prognostic factor in lung adenocarcinoma. Our findings will provide additional information on the significance of EFNAs and open new avenues for optimizing immunotherapy and accurately predicting prognosis.

## Methods

### Original data source and processing

The cancer genome atlas (TCGA) (https://portal.gdc.cancer.gov/) is a publicly accessible database with the Next Generation Sequencing (NGS) data of over 11,000 tumor samples from 33 tumors. Gene expression, copy number changes, methylation, and clinical information were obtained from these samples. The Illumina human methylation 450 platform was used to analyze methylation data. The Genotype-tissue expression (GTEx) database constitutes publicly available expression data from 53 normal tissue sites from nearly 1000 people. The GTEx and TCGA RNAseq data were obtained from UCSC Xena (https://xenabrowser.net/datapages/) [19] and used for the TCGA-GTEx combined analysis of unpaired samples. RNAseq data were obtained in Fragments Per Kilobase per Million (FPKM) format. TCGA and GTEx data were integrated and processed using R (version 4.1.0) and the limma package [20].

### Mutation and CNV analysis of EFNAs

The mutation frequency of EFNAs was evaluated in 33 types of tumors using the cBioPortal database (http://www.cbioportal.org, version v3.7.9), which is an open resource tool for analysis and visualization of multiple cancer genome data [21, 22]. This combined study includes 10,519 samples from the TCGA Pan-Cancer Atlas. Gene Set Cancer Analysis (GSCA) (http://bioinfo.life.hust.edu.cn/GSCA/#/) is a database that aggregates information on tumor genomes and immunogenomic gene sets [23]. We investigated the copy number variations (CNV) of the EFNA family in different cancers from the TCGA.

### Analysis of protein expression level

The Human Protein Atlas (HPA) database (https://www.proteinatlas.org/) is a free and open platform that provides scholars with valuable information on protein expression and localization in human tissues, cells, and organs [24, 25]. IHC was used to investigate the protein expression level of the EFNA family in various cancers using the HPA database. Immunocytochemistry (ICC) images were also obtained to detect and visualize EFNA5 protein in the human A431 epidermoid carcinoma cell line and U251MG glioblastoma cell line using antibodies specific to the target. Proteomic data were downloaded from the CPTAC database (https://proteomics.cancer.gov/data-portal) before examining the protein expression differences of EFNA1 and EFNA5 in cancer and para-cancer tissues.

### Protein interaction network construction

The String database (http://string-db.org) aggregates a colossal amount of publicly available information on protein–protein interactions [26]. The String database was used to construct the EFNAs protein–protein interaction (PPI) network. The interactive network was analyzed and observed using the igraph package (version 1.2.6) [27] and the ggraph package (version 2.0.5).

### GO and KEGG enrichment analysis

Proteins interacting with EFNAs were selected from the String database and analyzed for Gene Ontology (GO) and Kyoto Encyclopedia of Genes and Genomes (KEGG) enrichment. Meanwhile, the clusterProfiler package (version 3.14.3) [28] was used to explore significantly enriched Biological processes (BP) and KEGG pathway item (www.kegg.jp/kegg/kegg1.html), with all displayed items *p* < 0.05.

### Immune-related analysis

Data from TCGA on pan-cancer immune subtypes were obtained and the Kruskal test was used to compare the differences. TISIDB (http://cis.hku.hk/TISIDB/index.php) [29] was also used to evaluate the immune subtype in each cancer species, including C1 (wound healing), C2 (IFN-gamma dominant), C3 (inflammatory), C4 (lymphocyte depleted), C5 (immunological quiet), and C6 (TGF-beta dominant). The TCGA tumor samples were used to calculate the ImmuneScore, StromalScore, ESTIMATEScore, RNAss (RNA expression-based stemness score), DNAss (DNA methylation-based stemness score), tumor mutational burden (TMB), and microsatellite instability (MSI). These data were combined with EFNA family gene expression data to perform a Spearman correlation analysis. The xCell and TIMER algorithms with the immunedeconv package (version 2.0.3) [30] were used to explore the relationship between tumor-infiltrating immune cells (TICs) and EFNAs.

### Survival analysis

Clinical information of patients was obtained from the TCGA database. Thereafter, patients were divided into high and low expression groups based on the median of gene expression to analyze the overall survival (OS), disease-specific survival (DSS), disease-free interval (DFI), and progression-free interval (PFI) of patients. Also, the Kaplan–Meier Plotter database (https://kmplot.com/analysis/) [31] was used to examine the prognostic value of EFNAs mRNA expression in different cancers. Gene Expression Omnibus (GEO) survival data in Kaplan–Meier Plotter, including OS and recurrence-free survival (RFS) information, was a helpful supplement to the TCGA prognosis (RNA-seq). Furthermore, data from the GEO database (http://www.ncbi.nlm.nih.gov/geo/) are freely available to researchers, and three data sets were gathered (GSE50081, GSE72094, GSE68465) to investigate the relationship between gene expression and prognosis. The log-rank *p*-value and Hazard Ratio (HR) were calculated with 95% confidence intervals, and Log *p* < 0.05 was considered statistically significant. The survival package (version 3.2–10) was used for statistical analysis. The survminer package (version 0.4.9) was used for Kaplan–Meier curves and visualization. The rms package (version 6.2–0) was used to draw a nomogram that predicted the probability of death events and draw a calibration that reflected the actual probability and model probability under different conditions.

### Patients and tissue samples

A total of 13 pairs of lung adenocarcinoma and matched normal fresh frozen tissues were obtained through pneumonectomy at Harbin Medical University Cancer Hospital. This study was approved by the Ethics Committee of Harbin Medical University Cancer Hospital. Human lung tissue microarray (TMA) (No. HLugA180Su04) with 88 pairs of lung, para-cancer tissues and four additional lung adenocarcinoma tissues were purchased from Shanghai Outdo Biotech Company.

### Immunohistochemistry

H&E staining was used to determine the pathological type of all specimens before immunohistochemistry. Briefly, the microarray was baked at 63 °C for 60 min, dewaxed in xylene then hydrated in ethanol with different concentrations. Thereafter, the antigen was repaired using the antigen repair apparatus. The microarray was incubated overnight at 4℃ with the indicated primary EFNA5 antibody (1:100, Abmart, China). The secondary antibody was added and incubated at room temperature for 120 min. Eventually, the tissue microarray was counterstained for 1 min with hematoxylin and sealed. Two independent investigators assessed EFNA5 immunostaining. The percentage of protein-positive cells was calculated as follows: 0, 0%; 1, 1 ~ 25%; 2, 26 ~ 50%; 3, 51 ~ 75%; 4, > 75%. The intensity score was assigned a value of 0 (negative), 1 (weak), 2 (medium), and 3 (strong). The final staining score was calculated by adding the positive cell score and the intensity score (0–7). The EFNA5 staining was defined into two categories based on the staining score, i.e., low expression (0 ~ 4) and high expression (5 ~ 7) [32].

### Cell culture

The mRNA relative expression data of 10 NSCLC cell lines were obtained from the Cancer Cell Line Encyclopedia (CCLE) database (https://sites.broadinstitute.org/ccle/). All NSCLC cell lines and human lung bronchial epithelial cell line (HBE) were purchased from ATCC (Manassas, VA) and cultured in RPMI medium or DMEM high glucose supplemented with 10% fetal bovine serum, and Penicillin–Streptomycin Solution in a humidified 5% CO_2_ incubator at 37℃.

### Quantitative real-time PCR (RT-qPCR)

Total RNA extracted from lung cancer tissues by Trizol was reversely transcribed into cDNA using FastKing gDNA Dispelling RT SuperMix. Thereafter, RT-qPCR was performed using SYBR Green Kit (ROX; Roche, Toronto, ON, Canada).

### Western blot

Cultured cells were washed in PBS and lysed for 30 min on ice with RIPA buffer and a protease inhibitor cocktail. Protein concentrations were established using a BCA kit. Equal amounts of proteins were resolved on SDS-PAGE gel and transferred to the PVDF membrane. The membrane was incubated at 4℃ overnight with EFNA5 antibody (1:1000, Abmart, China) and β-tubulin antibody (Abways technology, China). On the second day, the second antibody was incubated for 1 h to bind to the target antibody.

### Cancer types

ACC, Adrenocortical carcinoma; BLCA, Bladder urothelial carcinoma; BRCA, Breast invasive carcinoma; CESC, Cervical squamous cell carcinoma and endocervical adenocarcinoma; CHOL, Cholangiocarcinoma; COAD, Colon adenocarcinoma; DLBC, Lymphoid neoplasm diffuse large B-cell lymphoma; ESCA, Esophageal carcinoma; GBM, Glioblastoma multiforme; HNSC, Head and neck squamous cell carcinoma; KICH, Kidney chromophobe; KIRC, Kidney renal clear cell carcinoma; KIRP, Kidney renal papillary cell carcinoma; LAML, Acute myeloid leukemia; LGG, Brain lower-grade glioma; LIHC, Liver hepatocellular carcinoma; LUAD, Lung adenocarcinoma; LUSC, Lung squamous cell carcinoma; MESO, Mesothelioma; OV, Ovarian serous cystadenocarcinoma; PAAD, Pancreatic adenocarcinoma; PCPG, Pheochromocytoma and paraganglioma; PRAD, Prostate adenocarcinoma; READ, Rectum adenocarcinoma; SARC, Sarcoma; SKCM, Skin cutaneous melanoma; STAD, Stomach adenocarcinoma; TGCT, Testicular germ cell tumors; THCA, Thyroid carcinoma; THYM, Thymoma; UCEC, Uterine corpus endometrial carcinoma; UCS, Uterine carcinosarcoma; UVM, Uveal melanoma.

### Statistical analysis

The Wilcoxon rank-sum test was used to compare two paired samples or ordered classification variables between groups. Spearman analysis was used to perform the correlation between gene expression. Clinical feature differences between groups were compared using the Chi-square test or Fisher's exact test. The area under the receiver operating characteristic (ROC) curve indicated the diagnostic value of EFNA family genes in lung adenocarcinoma (LUAD). The Cox regression model was used for univariate and multivariate analyses, and factors with a *p*-value less than 0.05 in univariate analysis were included in multivariate analysis. R software (version 4.1.0) was used for all statistical analyses. The ggplot2 package (version 3.3.3) was used for image visualization. A statistically significant difference was defined as a *p-*value less than 0.05.

## Results

### Co-expression, interaction and functional analysis of EFNAs

**Fig. 1:**
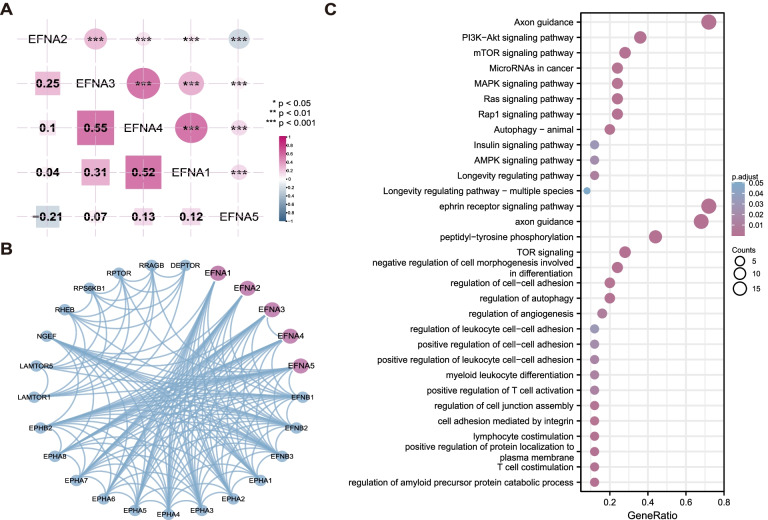
Genes highly correlated with EFNA family and the prediction of function. **A** Correlation of mRNA expression among EFNAs. **B** The protein–protein interaction network of EFNAs was evaluated by String. **C** GO and KEGG enrichment analysis by interacting protein molecules, and showing the significant enriched BP and KEGG pathway items. ^*^p < 0.05, ^**^p < 0.01, ^***^p < 0.001

The correlation of EFNAs co-expression at transcriptome level in pan-cancer was first examined, and the results suggested a strong correlation between EFNA1, EFNA3, and EFNA4 (Fig. 1A). Subsequently, the interacting proteins of EFNAs were extracted from the String database. Then, the interaction network was constructed as shown in Fig. 1B. Consequently, EFNAs were closely related to the EphA receptor family and the EFNB ligand family. In addition, we found significant protein interactions with DEPTOR, RRAGB, RPTOR, RPS6KB1, RHEB, NGEF, LAMTOR5, and LAMTOR1. All of the interacting molecules were thereafter subjected to GO and KEGG enrichment analyses. Results showed that EFNAs promoted immune function and cell adhesion besides the Eph-Ephine classical receptor-ligand in BP. Also, KEGG pathway results revealed that EFNAs were implicated in numerous tumor-related classical pathways including: the “PI3K-Akt signaling pathway”, “mTOR signaling pathway”, “MAPK signaling pathway” and “Ras signaling pathway” (Fig. 1C).

### mRNA and protein expression of EFNAs across cancer types

**Fig. 2:**
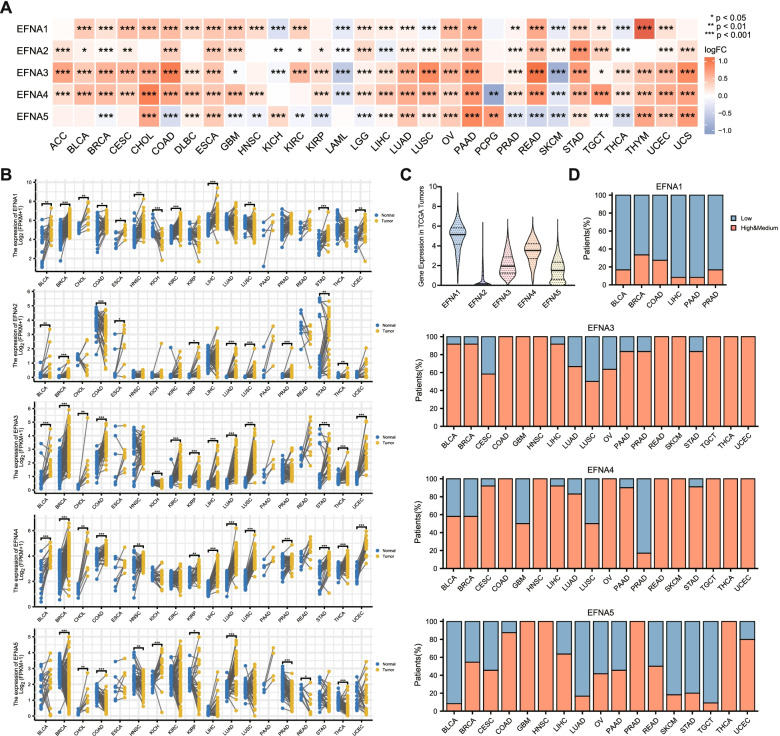
Expression level of EFNA family in pan-cancer. **A** The differential mRNA expression of EFNAs in tumor and normal tissues was evaluated by TCGA combined with GTEx. **B** The mRNA expression of EFNAs in cancer and para-cancer paired samples in TCGA. **C** The total mRNA relative expression of EFNAs in pan-cancer. **D** Protein expression pattern of EFNAs across various cancers in HPA. ^*^p < 0.05, ^**^p < 0.01, ^***^p < 0.001

The TCGA samples were combined with normal tissue samples in the GTEx database to examine mRNA level expression of EFNA family genes. In general, EFNAs were highly expressed in most tumor tissues in comparison to normal tissues, specifically in BLCA, CESC, CHOL, DLBC, ESCA, LUAD, OV, PAAD, STAD, UCEC, UCS and THYM (Fig. 2A). EFNAs remained up-regulated in BLCA, CESC, CHOL, ESCA, LUAD, PAAD, STAD, and UCEC following normal GTEx sample removal (Supplementary Fig. 1A). Then, using paired samples between tumor tissues and adjacent normal tissues from the TCGA, most EFNA family genes were up-regulated in BLCA, CHOL, LIHC, LUAD, STAD, and UCEC (Fig. 2B). Additionally, the relative mRNA expression level of EFNAs was examined in all TCGA tumor samples. EFNA1 had the highest expression, whereas EFNA2 had a relatively low overall expression (Fig. 2C).

Furthermore, the expression of EFNAs (except EFNA2) protein levels in the HPA database revealed moderate to strong staining of EFNA3/EFNA4, mainly in BLCA, BRCA, CESC, COAD, HNSC, LIHC, LUAD, OV, PAAD, READ, SKCM, STAD, TGCT, THCA and UCEC. Meanwhile, EFNA5 expression was moderate to high in COAD, GBM, HNSC, PRAD, THCA, and UCEC (Fig. 2D). Due to the small sample size in cancers, the high expression of EFNAs protein level in corresponding tumor tissues was not fully reflected. Furthermore, the protein expression levels of EFNA1 and EFNA5 in the CPTAC database were investigated using proteomic data. The results in the six cancer species for which data was available were completely consistent with the mRNA expression pattern in TCGA. For instance, EFNA1 was highly expressed in KIRC, LUAD, and UCEC compared to normal tissues, whereas EFNA5 was lowly expressed in GBM and KIRC and highly expressed in UCEC (Supplementary Fig. 1B).

### Genetic alterations variation and methylation of EFNAs across different cancers

**Fig. 3:**
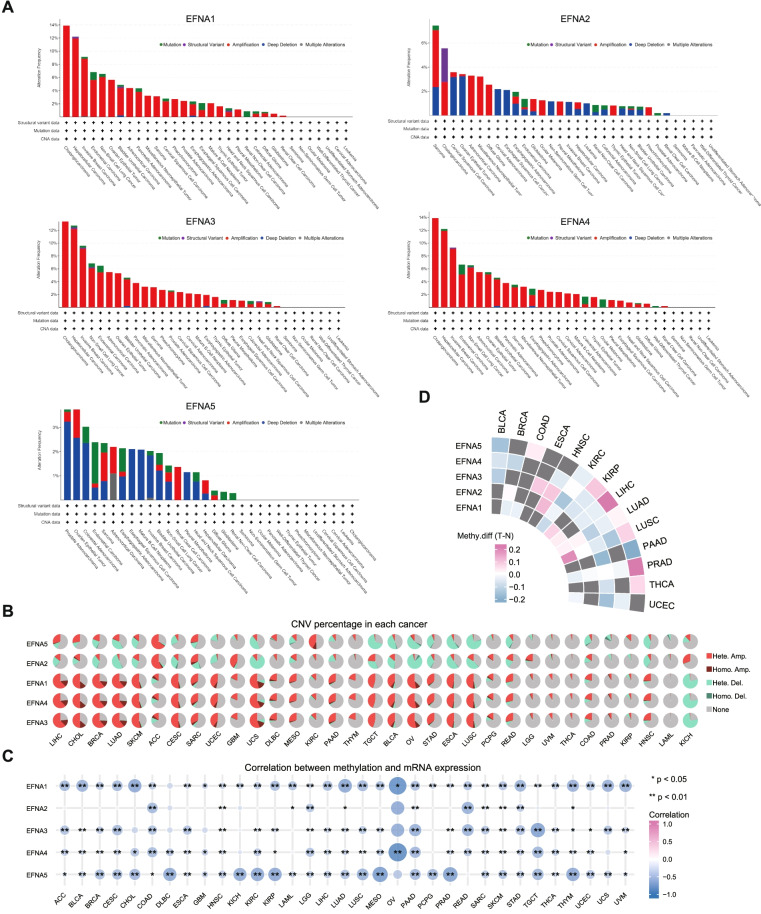
Variation and DNA methylation of EFNA family in pan-cancer. **A** Alteration frequency of EFNAs across various cancers was analyzed by cBioPortal. **B** Alterations of CNV of EFNAs in cancers were assessed from GSCA. **C** Correlations between DNA methylation and mRNA expression of EFNAs were determined by TCGA. **D** The methylation difference between tumor and para-cancer paired samples, and the gray dots indicated no statistical significance. ^*^p < 0.05, ^**^p < 0.01

Tumor development and immune tolerance are both affected by genomic and epigenetic changes. cBioPortal was used to investigate the mutation status of EFNAs in different tumors. The findings revealed that the variation frequency of EFNA1, EFNA3, and EFNA4 was relatively high. On the other hand, the variation in EFNAs was uncommon in leukemia, undifferentiated stomach adenocarcinoma, well-differentiated thyroid cancer, seminoma, and non-seminomatous germ cell tumor. Notably, all EFNAs had highly alteration in CHOL except for EFNA5 (Fig. 3A). The CNV percentage and the contribution of CNV to EFNAs expression were examined in each cancer. And a higher percentage of CNV was found in LIHC, CHOL, BRCA, LUAD, SKCM, CESC, UCS, TGCT, BLCA, OV, ESCA, and LUSC. The heterozygous/homozygous CNV (deletion/amplification) status of EFNA1, EFNA3, and EFNA4 had a similar alteration trend similar to EFNA2 and EFNA5 (Fig. 3B). As shown in Supplementary Fig. 2A, a positive correlation was noted between the expression of EFNA1, EFNA3, EFNA4, EFNA5 and CNV in most cancer types; however, EFNA2 expression was only weakly correlated with CNV. Generally, EFNA1, EFNA3, and EFNA4 had similar mutation patterns. Thereafter, we performed a correlation analysis between EFNA expression and DNA methylation to explore the effect of promoter region DNA methylation on EFNA expression (Fig. 3C). The mRNA expression of EFNA1 in OV and LUAD, EFNA3 in TGCT, EFNA4 in OV, and EFNA5 in MESO, PRAD, KIRP, THYM, KIRC, DLBC, and KICH was significantly suppressed by methylation (− 1 < cor <  − 0.6). The five most significant tumor data of each member of the EFNA family and methylation correlation were shown in Supplementary Fig. 2B. The methylation difference between tumor and normal samples was summarized in Fig. 3D. EFNA1 had higher methylation in LUSC, whereas EFNA5 had higher methylation in COAD, KIRP, PRAD, and THCA. Furthermore, EFNAs had lower methylation in most cancers than that in normal tissues. These findings suggest that abnormal EFNAs expression in some cancers could be attributed to genetic and DNA methylation changes.

### Association between EFNAs and immune infiltration in pan-cancer

**Fig. 4:**
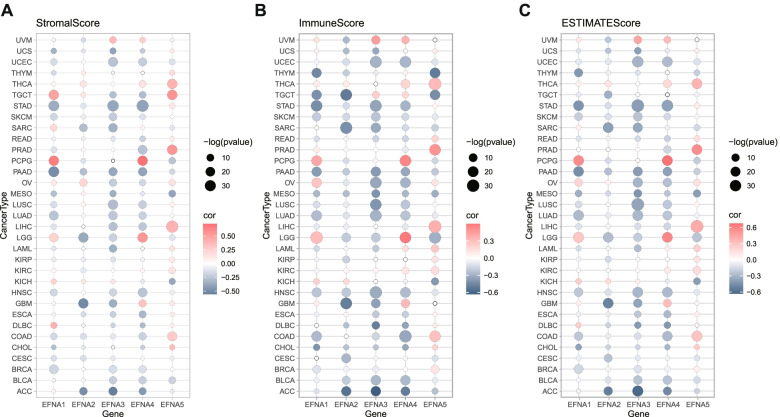
Correlation analysis between EFNA family expression and the tumor microenvironment in pan-cancer. **A-C** The correlation analysis of EFNAs with StromalScore, ImmuneScore, and ESTIMATEScore in pan-cancer

**Fig. 5:**
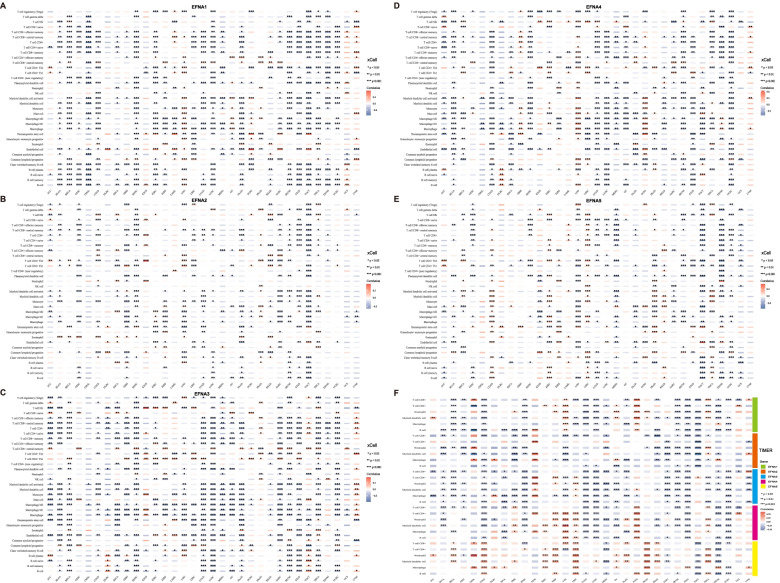
Tumor-infiltration immune cell of EFNA family. **A-E** Correlation analysis of EFNAs mRNA expression with immune/stromal cells from TCGA by xCell. **F** Correlation analysis of EFNAs mRNA expression with six major types of immune cells from TCGA by TIMER. ^*^p < 0.05, ^**^p < 0.01, ^***^p < 0.001

**Fig. 6:**
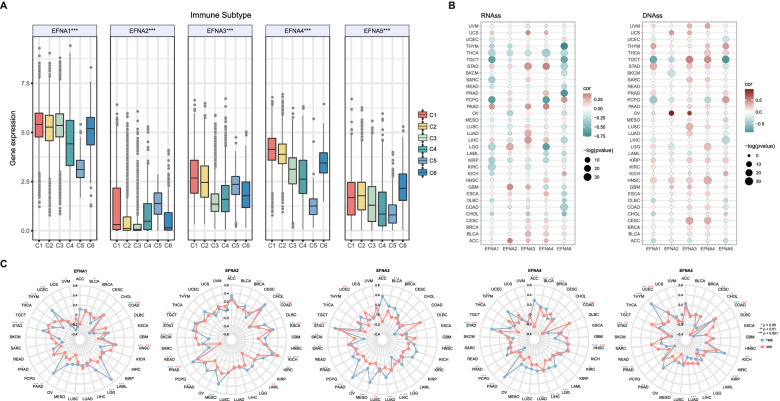
Correlation between EFNA family expression and immune subtypes, RNAss/DNAss and TMB/MSI. **A** EFNAs mRNA expression in six immune subtypes in pan-cancer. **B** Correlation analysis between expression of EFNAs and tumor stemness by RNAss/DNAss. **C** The correlation between expression of EFNAs and TMB/MSI by Radar diagram. ^*^p < 0.05, ^**^p < 0.01, ^***^p < 0.001

Evidence from functional enrichment and previous studies indicated that EFNAs regulate the tumor microenvironment (TME) [33]. Since stromal cells and immune cells are the primary effectors in TME, the relationship between EFNAs and stromal and immune scores were evaluated in pan-cancer (Fig. 4A and B). In the majority of cancers, EFNA1, EFNA2, EFNA3, and EFNA4 were suppressed by stromal and/or immune components, whereas EFNA5 demonstrated different trends in the stromal and immune scores across different cancers. Furthermore, the stromal and immune scores had a similar trend for one gene in most cancers. In TGCT, for instance, the stromal score of EFNA1 and EFNA5 in TGCT was inversely proportional to the immune score (Fig. 4A and B). Based on the overall ESTIMATEscore, EFNA1/2/3/4 negatively correlated with non-cancer components in the majority of cancer types (Fig. 4C). Therefore, the xCell approach was used to comprehensively evaluate EFNAs and immune infiltration, which was consistent with the ESTIMATEscore trend. EFNA1 negatively correlated with most of the immune cells in BRCA, CHOL, COAD, HNSC, LUAD, LUSC, MESO, PAAD, SKCM, STAD, and THYM (Fig. 5A); EFNA2 negatively correlated with the infiltrated immune cells in GBM, SARC and TGCT (Fig. 5B); EFNA3 negatively correlated with immune cells in nearly all cancers except UVM (Fig. 5C); EFNA4 positively correlated with various immune cells in GBM, LGG, PCPG and UVM, but negatively correlated with other cancers (Fig. 5D); EFNA5 positively correlated with several immune cells in COAD, KIRC, LIHC, PRAD, READ and THCA, yet negatively correlated with LUAD, MESO, TGCT, THYM, and UCEC (Fig. 5E). Using the TIMER algorithm, we also assessed the relationship between immune infiltration of major immune cells (including T cell, neutrophil, myeloid dendritic cell, and macrophage) and EFNAs. The results were in line with the xCell data presented above (Fig. 5F). Through immune subtypes, the highest expression level of EFNA1, EFNA3, and EFNA4 were observed in subtype C1; the highest expression level of EFNA2 in C5; the highest expression level of EFNA5 in subtype C6 (Fig. 6A). Furthermore, statistically significant data on the expression of EFNAs were obtained in each cancer subtype (showing the 6 most significant differences) (Supplementary Fig. 3). Based on RNA expression data, EFNA1 and EFNA5 were negatively correlated with the stem cell scores in most cancers, whereas EFNA2, EFNA3, and EFNA4 exhibited varying correlations with stem cell scores in different cancers. The higher the tumor stemness index score, the more active tumor stem cells are, and the lower the degree of differentiation. DNA methylation data showed that EFNA2 and EFNA3 positively correlated with tumor stemness in most cancers, whereas other family members revealed opposite results in various cancers (Fig. 6B). TMB and MSI are associated with the therapeutic effects and prognosis of cancer immunotherapy [34–36]. We evaluated the relationship between EFNAs mRNA expression and TMB/MSI (Fig. 6C). Consequently, EFNAs were strongly associated with TMB/MSI in a few cancers, specifically in COAD, STAD, PAAD, etc. Collectively, EFNAs could be potential molecules with immunotherapy biological function.

### Correlation between mRNA expression and prognostic value of EFNAs

**Fig. 7:**
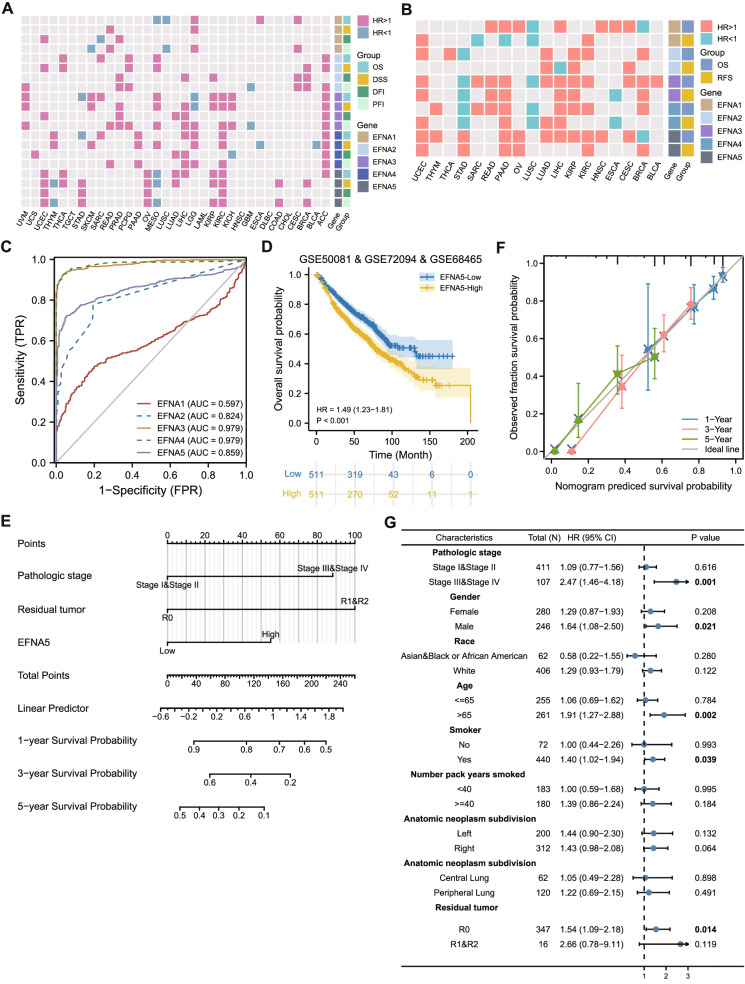
The clinical value of EFNA family in cancers. **A** EFNAs mRNA expression correlated with OS, DSS, DFI, and PFI from TCGA. **B** EFNAs mRNA expression correlated with OS and RFS in various cancers from GEO and other datasets. The prominently colored labels indicated *p* < 0.05. **C** Diagnostic ROC for EFNAs in LUAD and normal samples by TCGA combined with GTEx. **D** The overall survival curve of EFNA5 was analyzed integrated by the three LUAD data sets in GEO. **E** The nomogram for predicting 1 -, 3-and 5-year overall survival probability in LUAD patients. **F** Calibration of the nomogram for cox regression model and actual situation fitting analysis. **G** Univariate survival analysis of EFNA5 expression in subgroups with different characteristics

Based on TCGA, we discovered a link between EFNAs mRNA expression and patient survival prognosis (OS, DSS, DFI, PFI). The results showed that high EFNAs expression predicted a poor prognosis in most cancers. However, a few exceptions were found, including EFNA1 in LUSC and EFNA5 in THYM and MESO, which both played a protective role (Fig. 7A). We also provided a forest map of overall survival in TCGA (Supplementary Fig. 4). Using GEO and other projects survival data (OS, RFS) acquired from Kaplan–Meier Plotter, the prognostic trend of EFNAs was consistent with the TCGA in most cancers (Fig. 7B).

### Correlation between expression of EFNAs and the clinical features in LUAD

The findings suggested that EFNAs, specifically EFNA5, played an important role in LUAD. The ROC curve was used to estimate the diagnostic efficacy of EFNAs in LUAD tissues and normal samples. EFNA1 had low accuracy in predicting normal and tumor outcomes, (AUC = 0.597, CI = 0.560–0.634), whereas EFNA2 had a certain accuracy (AUC = 0.824, CI = 0.800–0.849). The prediction power of variable EFNA3 (AUC = 0.979, CI = 0.970–0.987) and variable EFNA4 (AUC = 0.979, CI = 0.969–0.989) was high, and that of variable EFNA5 was accurate (AUC = 0.859, CI = 0.834–0.885) (Fig. 7C). Table 1 showed the primary clinical features of LUAD in the TCGA database. The results indicated that EFNA2 expression correlated with tumor residue; the expression of EFNA3 and EFNA4 correlated with age; EFNA5 expression correlated with the number of pack-years smoked. Cox regression univariate analysis of EFNAs and clinical characteristics was used to predict the prognosis value of EFNAs. As a consequence, pathological stage, residual tumor, and the expression of EFNA2, EFNA3, and EFNA5 had significant prognostic significance in LUAD. The factors with significant statistical significance in univariate analysis (*p* < 0.05) were then included in multivariate analysis. The pathological stage (HR = 2.318, *p* < 0.001), tumor residue (HR = 2.475, *p* = 0.004) and EFNA5 (HR = 1.731, *p* = 0.001) were independent prognostic indicators of LUAD (Table 2). Given the significant prognostic effect of EFNA5 on LUAD, three LUAD GEO datasets were combined to evaluate the prognostic value of EFNA5. The results showed that those with high EFNA5 expression had a significantly worse prognosis (HR = 1.49, 95CI = 1.23–1.81, *p* < 0.001); this was consistent with the findings of the TCGA and Kaplan–Meier Plotter (Fig. 7D).

**Table 1:** The relation between EFNA1/2/3/4/5 expression and clinical characteristics in LUAD in TCGA

Characteristics	EFNA1 (*P* value)	EFNA2 (*P* value)	EFNA3 (*P* value)	EFNA4 *(P* value)	EFNA5 (*P* value)
Pathologic stage (I, II, III, IV)	0.476	0.348	0.642	0.940	0.251
Gender (Female, Male)	1.000	0.575	0.894	0.364	0.514
Race (Asian, Black or African American, White)	0.687	0.213	0.321	0.159	0.193
Age (≤ 65, > 65)	0.186	0.114	0.216	0.008	0.292
Age, median (IQR)	0.213	0.176	0.037	0.019	0.317
Residual tumor (R0, R1, R2)	0.918	0.005	1.000	0.219	0.558
Anatomic neoplasm subdivision1 (Left, Right)	1.000	0.061	0.196	1.000	0.427
Anatomic neoplasm subdivision2 (Central, Peripheral)	0.909	0.833	0.765	0.466	0.675
Number pack years smoked (< 40, ≥ 40) Smoker (No, Yes)	0.434 0.682	0.232 0.219	0.234 1.000	0.962 0.986	0.011 1.000

**Table 2:** Univariate and multivariate analyses of overall survival in patients with LUAD in TCGA

Characteristics	Total(N)	Univariate analysis		Multivariate analysis	
		Hazard ratio (95% CI)	*P* value	Hazard ratio (95% CI)	*P* value
Pathologic stage (Stage III&IV vs. Stage I&II)	518	2.664 (1.960–3.621)	** < 0.001**	2.318 (1.602–3.355)	** < 0.001**
Gender (Male vs. Female)	526	1.070 (0.803–1.426)	0.642		
Race (White vs. Asian&Black or African American)	468	1.475 (0.902–2.411)	0.121		
Age (> 65 vs. ≤ 65)	516	1.223 (0.916–1.635)	0.172		
Smoker (Yes vs. No)	512	0.894 (0.592–1.348)	0.591		
Number pack years smoked (≥ 40 vs. < 40)	363	1.004 (0.997–1.011)	0.223		
Anatomic neoplasm subdivision (Right vs. Left)	512	1.037 (0.770–1.397)	0.810		
Anatomic neoplasm subdivision (Peripheral Lung vs. Central Lung)	182	0.913 (0.570–1.463)	0.706		
Residual tumor (R1&R2 vs. R0)	363	3.879 (2.169–6.936)	** < 0.001**	2.475 (1.341–4.570)	**0.004**
EFNA1 (High vs. Low)	526	1.017 (0.764–1.355)	0.906		
EFNA2 (High vs. Low)	526	1.691 (1.262–2.266)	** < 0.001**	1.247 (0.881–1.766)	0.213
EFNA3 (High vs. Low)	526	1.429 (1.071–1.908)	**0.015**	1.279 (0.911–1.795)	0.155
EFNA4 (High vs. Low)	526	0.990 (0.744–1.318)	0.947		
EFNA5 (High vs. Low)	526	1.601 (1.198–2.141)	**0.001**	1.731 (1.238–2.420)	**0.001**

Using cox regression analysis, we developed a prognostic model in LUAD that included pathological stage, tumor residue, and EFNA5 expression. Thereafter, we presented the prediction of 1-year, 3-year, and 5-year OS in form of a nomogram (Fig. 7E). By drawing the fitting situation of the actual probability and the probability predicted by the model under different circumstances in the graph, the model had the best predictive effect on the 1-year survival rate of the actual result, and the fitting degree of 3-year and 5-year survival rate was also accurate. (Fig. 7F). Moreover, we investigated the prognostic value of EFNA5 in a subset of LUAD patients (Fig. 7G). Consequently, EFNA5 correlated with poor prognosis for OS in patients with advanced pathologic stage (stage III & IV) (HR = 2.47, *p* = 0.001), male (HR = 1.64, *p* = 0.021), age over 65 (HR = 1.91, *p* = 0.002), smoking (HR = 1.40, *p* = 0.039), R0 resection (HR = 1.54, *p* = 0.014).

### Preliminary experimental verification of characteristics of EFNA5

**Fig. 8:**
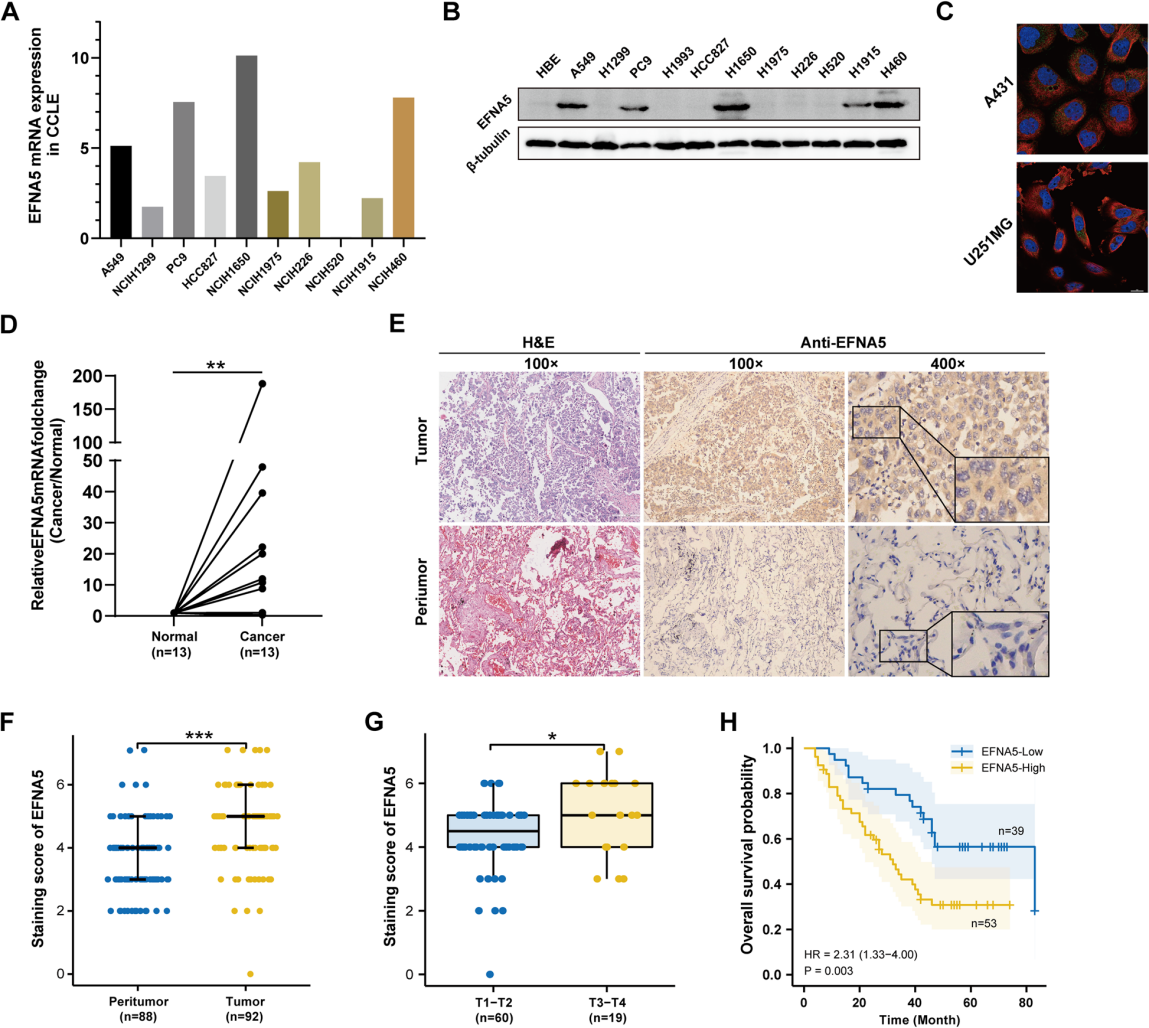
Preliminary experimental verification of characteristics of EFNA5. **A **EFNA5 mRNA expression level in NSCLC cell lines from CCLE. **B** The expression level of EFNA5 in a normal lung bronchial epithelial cell line (HBE) and NSCLC cell lines was determined by Western Blot. **C** Immunocytochemistry for determining the subcellular location of EFNA5 in A431 and U251MG cell lines by HPA. EFNA5 localized to the membrane, cytosol, and vesicles (green). Microtubules are stained in red and the nucleus in blue (DAPI). **D** The mRNA relative expression level of EFNA5 on 13 LUAD and para-cancer paired tissues was determined by RT-qPCR. **F** Representative images of TMA stained with H&E and IHC for EFNA5. **G** EFNA5 expression levels in LUAD and peritumoral tissues determined in TMA. **H** EFNA5 staining score increased significantly with the characteristics of the primary tumor. **I** Overall survival analysis of 92 LUAD patients according to EFNA5 expression. ^*^p < 0.05, ^**^p < 0.01, ^***^p < 0.001

We targeted the expression of EFNA5 in NSCLC, and its clinical significance as well as prognostic value to confirm the above findings. Using the CCLE database, we first looked at EFNA5 expression at the transcriptome level in NSCLC cell lines (Fig. 8A). Subsequently, EFNA5 expression was confirmed at the protein level in cell lines using western blot assay. The findings showed that EFNA5 was highly expressed in A549, PC9, H1650, and H460 cell lines agreeing with the transcriptional results (Fig. 8B). The HPA database was used to determine the subcellular localization of EFNA5 protein in A431 and U251MG cells using ICC; the results indicated that EFNA5 was primarily expressed in the cytosol and plasma membrane (Fig. 8C). Further, RT-qPCR was used to determine EFNA5 expression in 13 pairs of LUAD and peritumoral tissues. The findings showed higher EFNA5 expression in most tumor tissues (Fig. 8D). To confirm the dysregulation of EFNA5, histochemical analysis was performed on 180 paraffin-embedded lung adenocarcinoma and peritumoral tissues (Fig. 8E). In LUAD and peritumoral tissues, the positive rate of EFNA5 was 57.61% vs. 22.74% respectively (Fig. 8F). Moreover, we discovered significantly high IHC scores for EFNA5 in cases with high T stages (Fig. 8G). Patients with high EFNA5 expression had shorter OS (HR = 2.31, *p* = 0.003) (Fig. 8H). Supplementary Table 1 shows a comprehensive analysis of the differences between groups based on EFNA5 expression. Univariate and multivariate analyses showed that pathological stage and EFNA5 expression were independent prognostic factors of resected lung adenocarcinoma, correlating with the TCGA database results (Supplementary Table 2).

## Discussion

This study explored the potential role of EFNA family members in pan-cancer. EFNA1-5 showed significant differences in mRNA and protein levels across cancer types. Previous evidence indicated that the expression of EFNAs varied significantly between tumor and non-neoplastic diseases [37, 38]. CNV has been identified as the genetic basis for somatic chromosomal deletion and replication development in tumors, suggesting that it could be a breeding ground for acquired changes in cancer cells [39]. Moreover, epigenetic mechanisms regulate the expression of EFNAs, and DNA methylation alters gene expression without causing changes in gene sequence [40]. EFNAs changes were investigated at genomic and epigenetic levels, indicating that CNV and promoter DNA methylation modulates EFNAs expressions in various cancers. We hypothesized that tumor cell evolution potentially mediates changes in EFNAs expression to some extent, and these mutations have previously been linked to malignant tumor behavior and carcinogenesis.

Based on GO and KEGG enrichment analyses, the EFNA family is linked to oncogenic functions and pathways (such as PI3K-Akt, mTOR, MAPK, autophagy, and angiogenesis). Previous studies indicate that EFNA1is involved in invasion, angiogenesis, and malignant phenotypes [32, 41, 42]. Literature evidence shows that EFNA2 promotes EMT and increases angiogenesis in prostate cancer [43]. EFNA3 helps in proliferation, invasion of peripheral nerve sheath tumors, and tumor angiogenesis [44, 45]. EFNA4 potentially promotes hepatocellular carcinoma invasion and migration via the GSK3β signaling pathway [15], and is involved in the invasion of glioma via Akt signaling [46]. Furthermore, EFNA5 promotes the malignant progression of pancreatic cancer [47] but played a tumor suppressor role in glioma by inhibiting EGFR [48], tumor invasiveness, and metastasis in hepatoma and colorectal cancer [49, 50]. Whether EFNAs act as an oncogene or as a tumor suppressor in different cancers remains unclear, and accurate underlying mechanisms are unknown. Additionally, EFNAs were linked to immune regulation, providing a reference for investigating the role of EFNAs in TME.

The complex TME is made up of tumor cells, immune cells, stromal cells, and extracellular components, all of which perform varied roles. Some of these roles contribute to the establishment of an immunosuppressive environment, affecting various treatment responses to tumors [51]. In this work, we evaluated the stromal score, immune score, and total ESTIMATE score of EFNAs in pan-cancer, and found that EFNA family members inhibit immune function in various tumors. Previous studies focused on killing immune cells including effectors T cells, NK cells, Dendritic cells, M1 polarized macrophages, N1 polarized neutrophils, and suppressive immune cells, including regulatory T cells (Tregs) and myeloid-derived suppressor cells (MDSCs). However, the role of B cells in TME is controversial [52]. Furthermore, we discovered that EFNAs linked to suppressive immune components in various tumors; this explains why cancer patients expressing EFNAs have a poor prognosis. Furthermore, the different correlations of EFNAs in immune subtypes could contribute to the difference in cancer prognosis. Cancer stem cells, also known as tumor-propagating cells, can self-renew and remain undifferentiated, rendering them resistant to chemoradiotherapy [53]. RNAss based on mRNA expression and DNAss based on DNA methylation were used to determine the stemness of cancers [54]. We found a significant correlation of EFNAs with stem cell scores in 33 cancers. Given the RNA expression (RNAss), the expression of EFNAs negatively correlated with stem cell score in THCA and KIRP, indicating that an increase in EFNA expression level is associated with a decrease in tumor stem cell number and an increase in tumor differentiation. Besides, based on DNA methylation (DNAss), the expression of EFNAs in SARC, HNSC, and BRCA positively correlated with stem cell score, implying that the higher the EFNA expression level, the more tumor stem cell characteristics, and the poorer the differentiation. EFNA4 regulates stem cell properties in glioma [46], whereas EFNA5 regulates the phenotype of breast cancer stem cell-like cells [55]. In TME, the Eph receptor acts as an immunosuppressor by modulating tumor interactions with innate and adaptive immune cells [10, 33]. Cellular interaction in breast cancer is based on EphA10 and EFNA3 expression, which binds to PD1 on T cells to inhibit immune activity and promote tumor tolerance [56]. The interaction of information between tumor cells via Ephs/ephrins suggests that EFNA is a potential immunotherapy target.

Recent evidence shows that TMB can be used as a marker for immune checkpoint inhibitors in various cancers. The high mutation burden in tumors promotes the formation of neoantigens, making tumors more immunogenic and improving immunotherapy response [34, 57, 58]. MSI is caused by the deletion or insertion of base pairs in the microsatellite region and is a potential target for predicting the efficacy of tumor immunotherapy. Tumors with mismatch-repair deficiency (dMMR) and MSI-H respond better to immunotherapy [59]. In the present study, EFNA1/2 expression in COAD was negatively correlated with both TMB and MSI, whereas EFNA5 was positively correlated with both. Furthermore, EFNA1/2/3/4 positively correlated with the two targets in STAD, whereas EFNA5 negatively correlated with them. The combination of TMB and MSI analyses is critical in selecting patients suitable for immunotherapy in numerous cancers [60].

The different Ephs/Ephrins members have tumor-promoting or suppressive properties, making them potential therapeutic targets. Therapy agents that inhibit Ephs/Ephrins are emerging future treatment options. Clinical trials have provided novel therapeutic options as targeted therapies. They include antibody inhibitors blocking EphA2 in esophageal and gastric cancer [61], antibody blockers against EphA3 in hematologic malignancies [62], protein complex blockers against EFNB2 in solid and hematologic cancers [63], and an EFNA4 monoclonal antibody conjugate, the ADC (antibody–drug conjugate) drug PF-06647263 [64].

Furthermore, we discovered that EFNAs had a significant difference in prognosis among cancers and EFNA2/3/4/5 had potential diagnostic value in lung adenocarcinoma. Evidence from a related study suggests that exosomal EFNA2 is a diagnostic biomarker for prostate cancer [65]. Additionally, based on the TCGA and GEO data, EFNAs play a negative prognostic role in most cancers and suggest a better prognosis in a small number of tumor types. The variable prognostic significance of a particular factor in various cancers could be explained by tumor heterogeneity, a major challenge for tumor treatment [66, 67]. Previous research revealed that EphA2-positive with EFNA1-negative glioma patients had shorter OS and PFS [68]. EFNA1 and EphA2 co-expression in BRCA is associated with disease recurrence [69]. EFNA1 overexpression is linked to low DFS of hepatoma [70]. EFNA4 expression is linked to poor prognosis in hepatoma [15]. High EFNA5 expression is associated with lower OS in ovarian carcinoma [71] and pancreatic cancer [47] but associated with a better prognosis in prostate cancer [17], hepatoma [49], and glioblastoma [18]. On the other hand, the clinical value of EFNA5 in LUAD has received limited research attention. In this work, EFNA5 levels in the TCGA and GEO databases were linked to a shorter overall survival of patients with LUAD. The mRNA and protein expression levels of patient samples were demonstrated, showing that EFNA5 is an independent adverse prognostic factor in LUAD related to the T stage. EFNA5 exerts tumor-promoting and suppressive effects in various tumors, which is the center of this work. Therefore, further experimental studies should be performed to unravel the mechanism by which EFNA5 mediates the malignant function of LUAD cells, resulting in a poor prognosis for patients.

This study has compelling limitations. First, we examined the role of EFNA family members in pan-cancer from a broad perspective; however, additional experimental and clinical validation is necessary for molecules with potential research value. Moreover, the corresponding mechanism was not depicted in its entirety. Thirdly, it is difficult to predict and evaluate the efficacy of immunotherapy since expression data of the immunotherapy patients are unavailable. These issues will be resolved further in our subsequent research.

## Conclusion

**Fig. 9:**
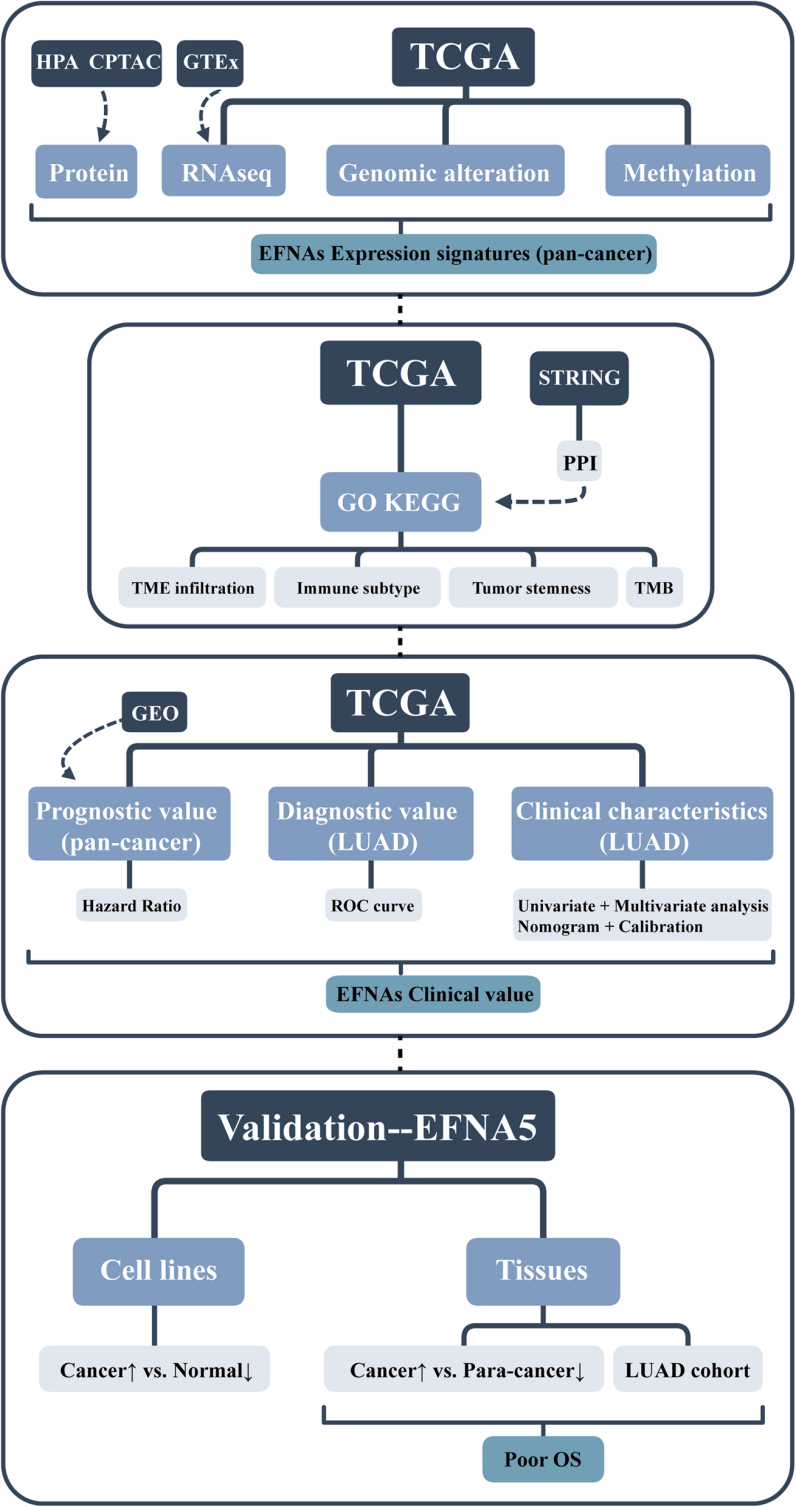
A detailed flow chart shows the analysis explanation of this study

In conclusion, we used a multi-omics approach to explore the mRNA and protein expression, potential function, immune infiltration, clinical features, and prognostic value of the EFNA family. Further, we confirmed that EFNA5 expression is associated with poor prognosis in LUAD (Fig. 9). These findings confirm the significance of EFNAs expression in predicting tumor prognosis involving in the immune microenvironment, and provide key references for future research.

## Supplementary Information

**Additional file 1. **

## Data Availability

The data that support this study are available from the corresponding authors through reasonable request.

## Abbreviations

CNV: Copy number variations
DFI: Disease free interval
DSS: Disease specific survival
FPKM: Fragments Per Kilobase per Million
GO: Gene ontology
IHC: Immunohistochemistry
KEGG: Kyoto Encyclopedia of Genes and Genomes
MSI: Microsatellite instability
NSCLC: Non-small cell lung cancer
OS: Overall survival
PFI: Progression free interval
PPI: Protein–protein interaction
RFS: Recurrence free survival
RTKs: Receptor tyrosine kinases
TICs: Tumor infiltration cells
TMB: Tumor mutation burden
TME: Tumor microenvironment
