# Supplementary material for: Expression characteristic, immune signature, and prognosis value of EFNA family identified by multi-omics integrative analysis in pan-cancer
Source: BMC Cancer. 2022 Aug 10;22:871. doi: 10.1186/s12885-022-09951-0 (PMC9364540; doi:10.1186/s12885-022-09951-0)
Supplement: Supplementary file 1 — Additional file 1. [file 12885_2022_9951_MOESM1_ESM.pdf]

Supplementary Files

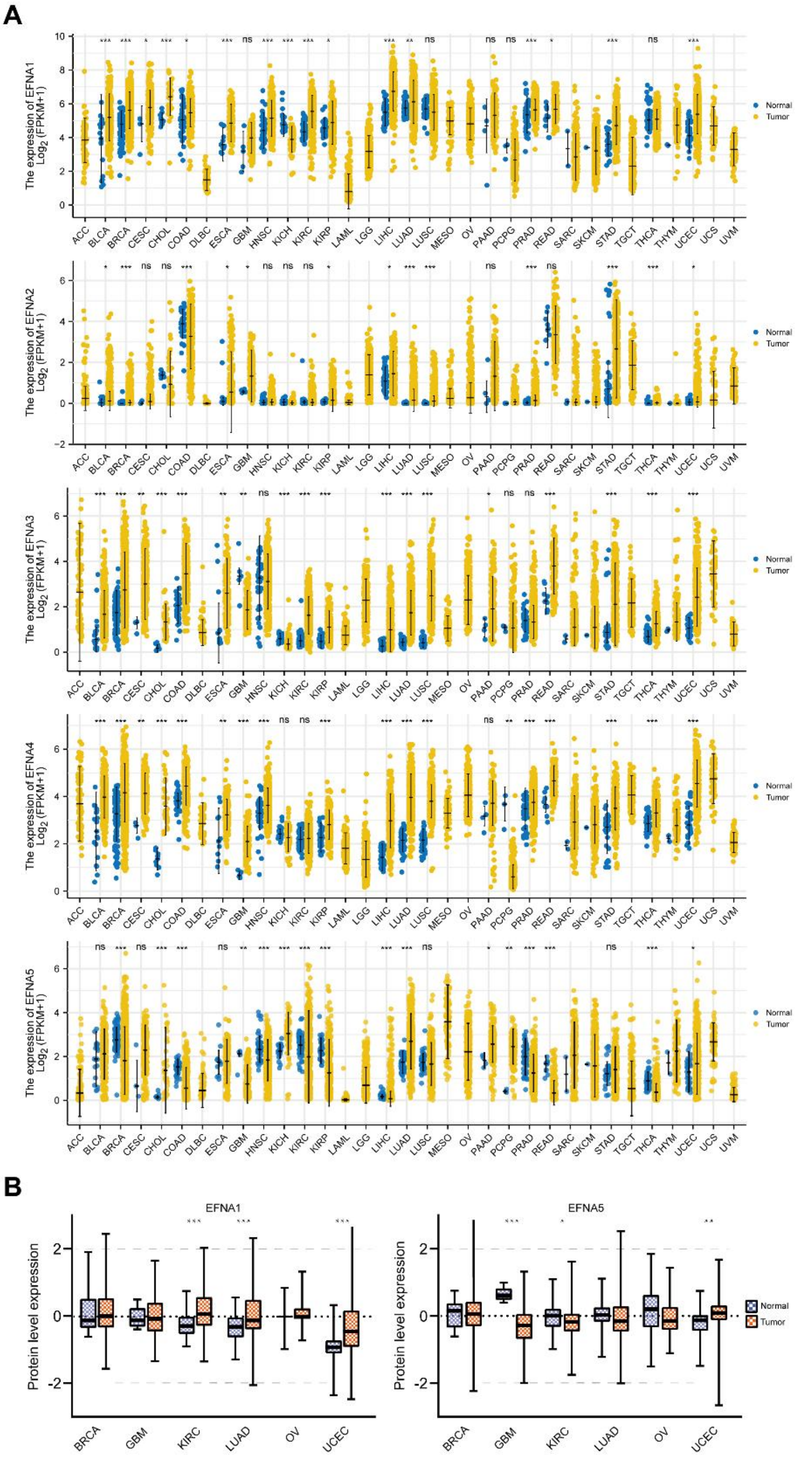

Fig. S1 Expression of EFNA family in tumor and normal tissues **(A)** The mRNA expression of EFNAs was evaluated across 33 cancers from TCGA. **(B)** The protein expression of EFNA1 and EFNA5 was evaluated from CPTAC. $^{*}p < 0.05$, $^{**}p < 0.01$, $^{***}p < 0.001$.

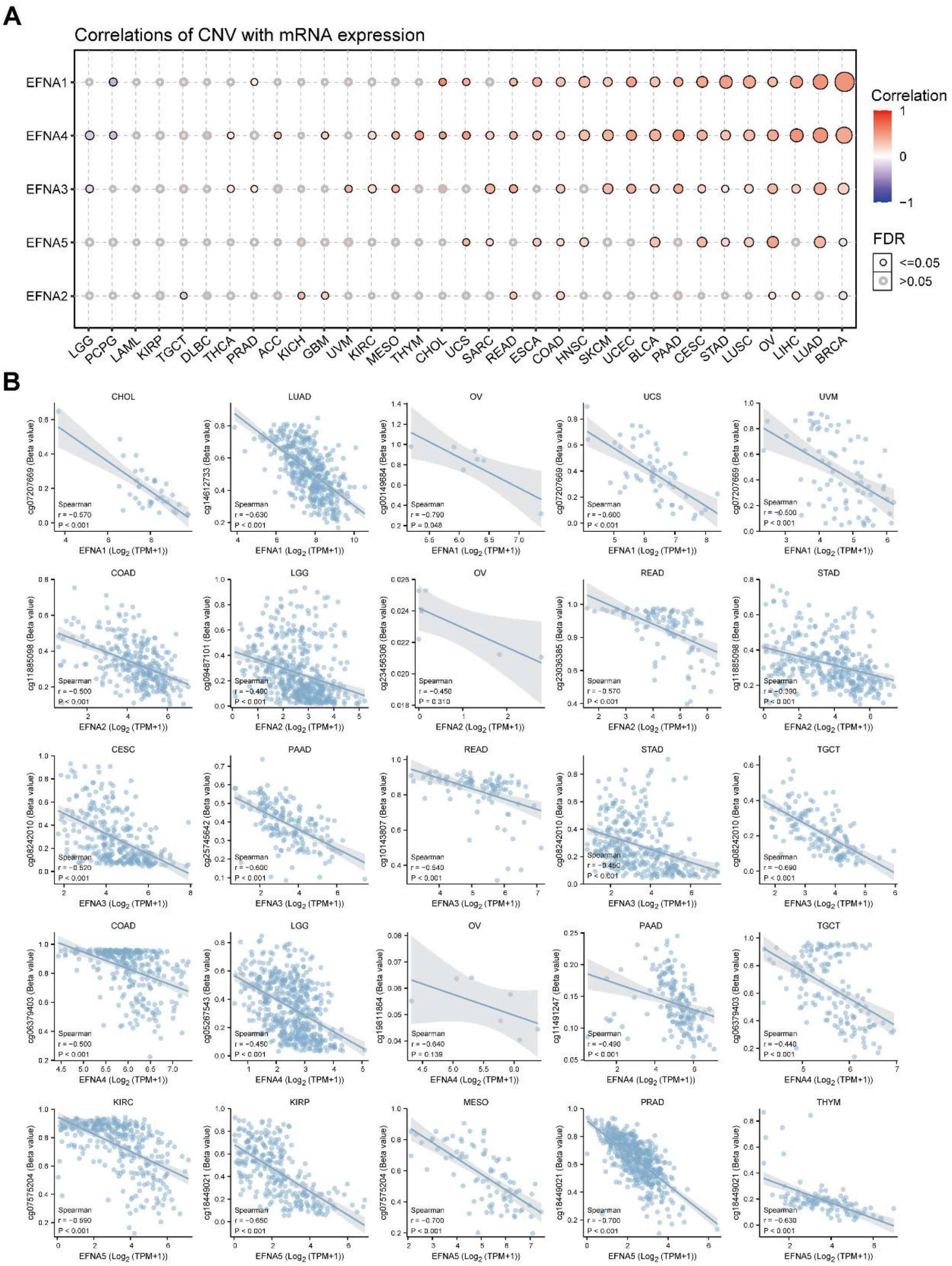

Fig. S2 CNA and DNA methylation of EFNA family in human cancers. **(A)** The correlation between EFNAs mRNA expression and CNV from TCGA. **(B)** The top 5 negative correlation between EFNAs mRNA expression and DNA methylation was demonstrated by TCGA.

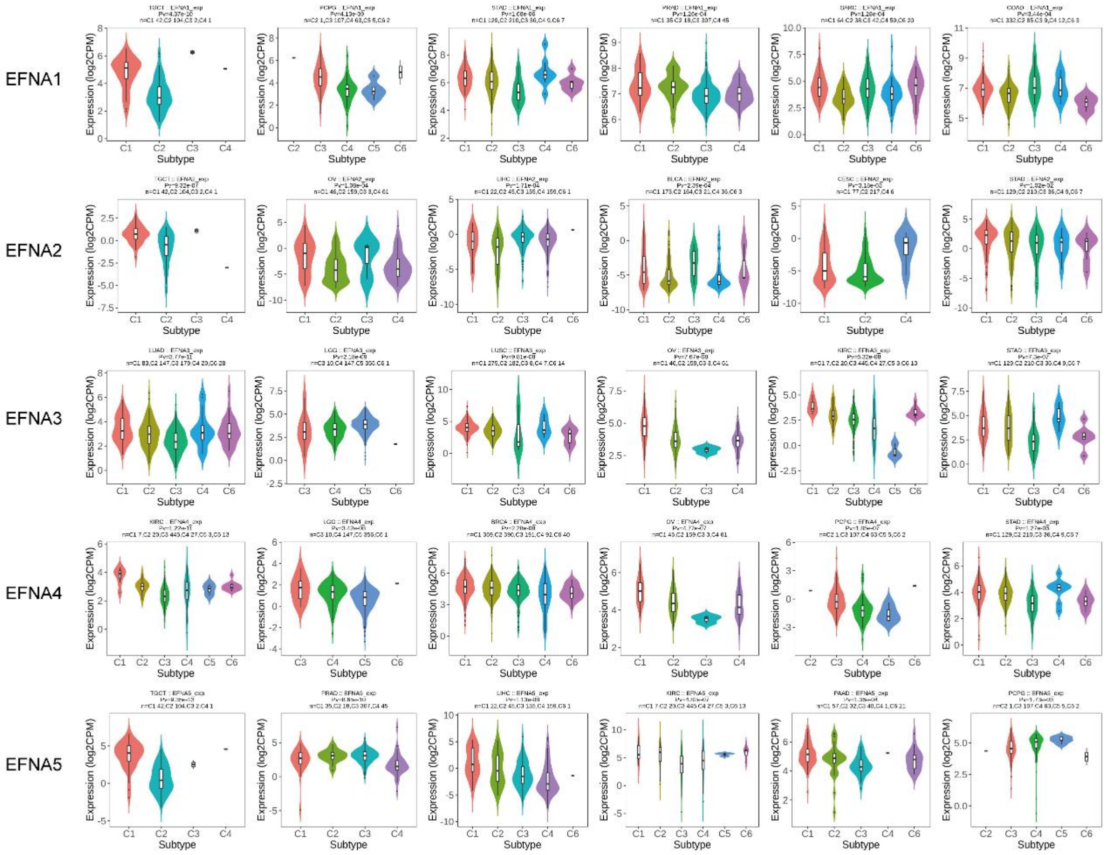

Fig. S3 EFNA mRNA expression in different immune subtypes through TISIDB. Showing the five most statistically significant cancer types for each gene.

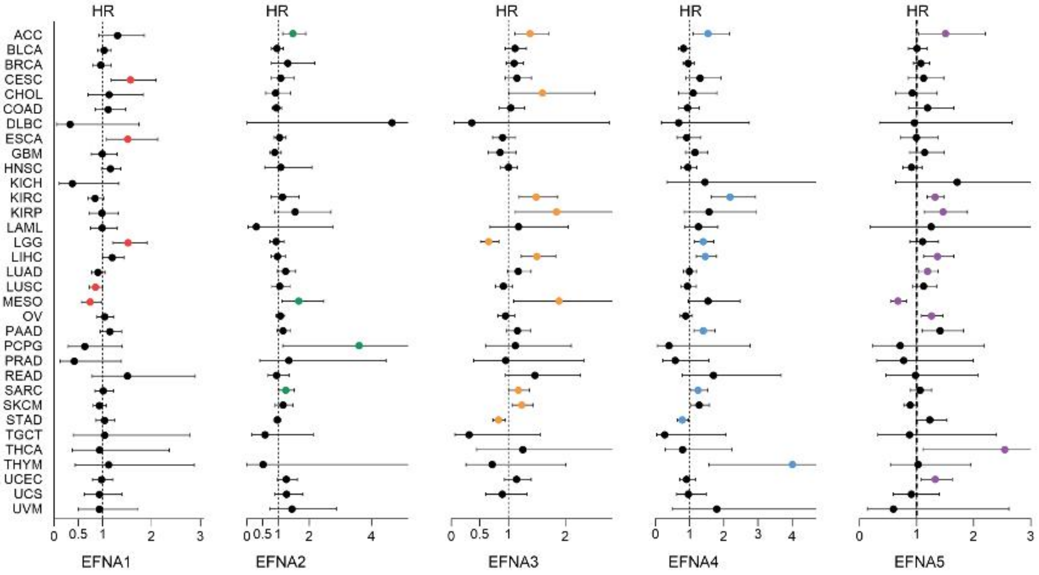

Fig. S4 Overall survival prognosis forest map of EFNA family genes in TCGA. The prominently colored labels indicated $p < 0.05$.

Table S1. The relation between EFNA5 expression and clinical characteristics in LUAD in TMA

Characteristics	EFNA5-High	EFNA5-Low	P
n	53	39	
T stage, n (%)			0.309
T1-T2	31 (39.2%)	29 (36.7%)	
T3-T4	13 (16.5%)	6 (7.6%)	
N stage, n (%)			0.841
N0	19 (27.1%)	15 (21.4%)	
N1-N3	22 (31.4%)	14 (20%)	
Pathologic stage, n (%)			1.000
I-II	24 (38.7%)	17 (27.4%)	
III-IV	12 (19.4%)	9 (14.5%)	
Gender, n (%)			0.635
Female	22 (23.9%)	19 (20.7%)	
Male	31 (33.7%)	20 (21.7%)	
Age, n (%)			0.218
≤ 65	30 (32.6%)	27 (29.3%)	
> 65	23 (25.0%)	12 (13.0%)	
Anatomic neoplasm subdivision, n (%)			0.334
Left	21 (23.6%)	11 (12.4%)	
Right	30 (33.7%)	27 (30.3%)	

Table S2. Univariate and multivariate analyses of overall survival in patients with LUAD in TMA

Characteristics	Total(N)	Univariate analysis		Multivariate analysis	
		Hazard ratio (95% CI)	P value	Hazard ratio (95% CI)	P value
Pathologic stage (Stage III&IV vs. Stage I&II)	62	3.096 (1.500-5.991)	**0.001**	3.044 (1.578-5.874)	**0.001**
Gender (Male vs. Female)	92	1.030 (0.782-1.356)	0.836		
Age (>65 vs. ≤ 65)	92	1.541 (0.884-2.687)	0.127		
Anatomic neoplasm subdivision (Right vs. Left)	89	1.018 (0.565-1.833)	0.953		
EFNA5 (High vs. Low)	92	2.399 (1.317-4.369)	**0.004**	1.408 (1.006-1.971)	**0.046**

Primer sequences of EFNA5 are as follows:

GAPDH-F	CACCCACTCCACCTTTGA
GAPDH-R	ACCACCCTGTTGCTGTAGCCA
EFNA5-F	GAGGACTCCGTCCCAGAAGAT
EFNA5-R	TTGGAGAGTGAGGCCGGTTA

Figure 8B. The full blots with membrane edges. (Multiple exposure images)

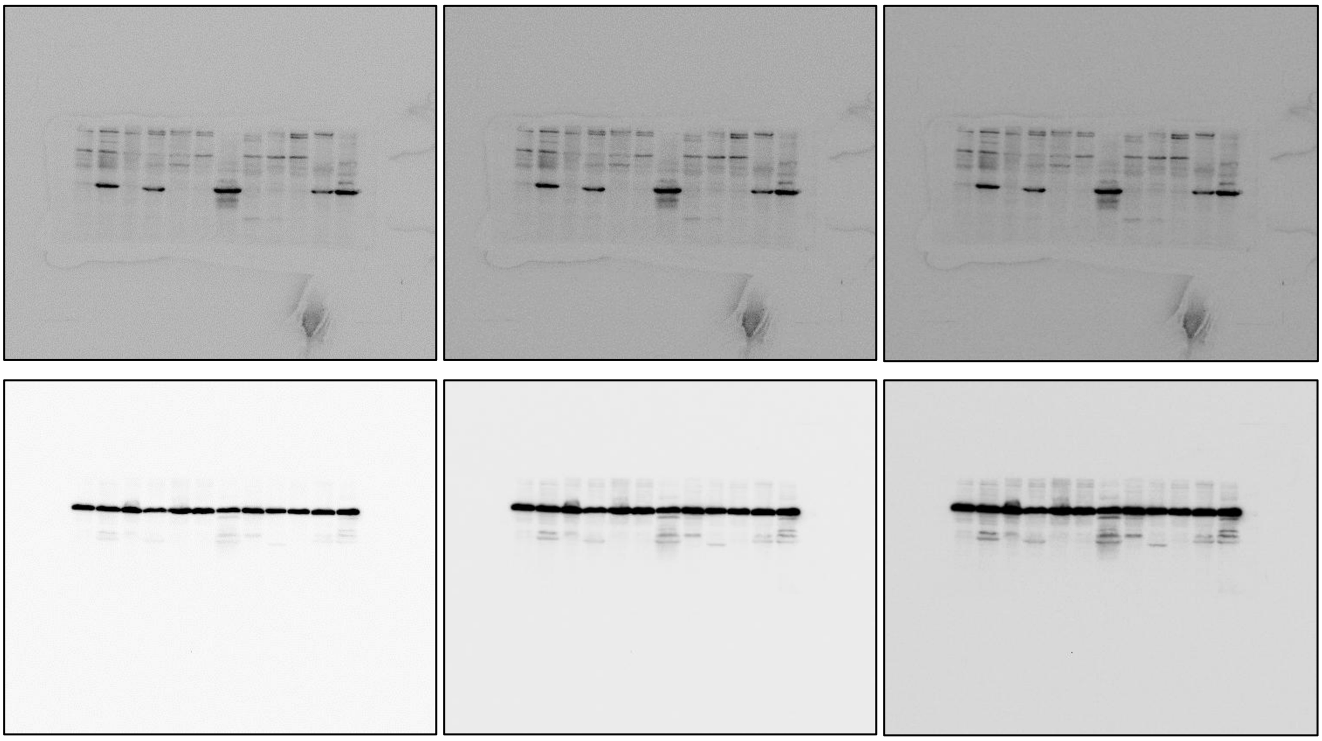

EFNA5

β-tubulin
